# HIV Testing in 50 Local Jurisdictions Accounting for the Majority of New HIV Diagnoses and Seven States with Disproportionate Occurrence of HIV in Rural Areas, 2016–2017

**DOI:** 10.15585/mmwr.mm6825a2

**Published:** 2019-06-28

**Authors:** Marc A. Pitasi, Kevin P. Delaney, John T. Brooks, Elizabeth A. DiNenno, Shacara D. Johnson, Joseph Prejean

**Affiliations:** 1 Division of HIV/AIDS Prevention, National Center for HIV/AIDS, Viral Hepatitis, STD, and TB Prevention, CDC.

Since 2006, CDC has recommended universal screening for human immunodeficiency virus (HIV) infection at least once in health care settings and at least annual rescreening of persons at increased risk for infection (*1*,*2*), but data from national surveys and HIV surveillance demonstrate that these recommendations have not been fully implemented (*3*,*4*). The national Ending the HIV Epidemic initiative* is intended to reduce the number of new infections by 90% from 2020 to 2030. The initiative focuses first on 50 local jurisdictions (48 counties, the District of Columbia, and San Juan, Puerto Rico) where the majority of new diagnoses of HIV infection in 2016 and 2017 were concentrated and seven states with a disproportionate occurrence of HIV in rural areas relative to other states (i.e., states with at least 75 reported HIV diagnoses in rural areas that accounted for ≥10% of all diagnoses in the state).^†^ This initial geographic focus will be followed by wider implementation of the initiative within the United States. An important goal of the initiative is the timely identification of all persons with HIV infection as soon as possible after infection (*5*). CDC analyzed data from the Behavioral Risk Factor Surveillance System (BRFSS)^§^ to assess the percentage of adults tested for HIV in the United States nationwide (38.9%), in the 50 local jurisdictions (46.9%), and in the seven states (35.5%). Testing percentages varied widely by jurisdiction but were suboptimal and generally low in jurisdictions with low rates of diagnosis of HIV infection. To achieve national goals and end the HIV epidemic in the United States, strategies must be tailored to meet local needs. Novel screening approaches might be needed to reach segments of the population that have never been tested for HIV.

BRFSS is an annual cellular and landline telephone survey of the noninstitutionalized U.S. population aged ≥18 years. The median response rate among all participating states and territories was 47.1% (range = 30.7%–65.0%) in 2016^¶^ and 45.9% (range = 30.6%–64.1%) in 2017.** Respondents were asked whether they had ever been tested for HIV outside of blood donation; those who answered “yes” were asked for the month and year of their most recent test. Respondents were also asked whether any of the following HIV risk–related situations applied to them in the past year: injected drugs that were not prescribed, received treatment for a sexually transmitted disease, exchanged money or drugs for sex, had anal sex without a condom, or had four or more sex partners. Those who answered “yes” to this question were considered to have reported recent HIV risk.

Data collected in 2016 and 2017 were pooled and used to estimate the percentage and corresponding 95% confidence intervals (CIs) of ever testing for HIV and testing for HIV in the past year overall and for each of the 57 jurisdictions. Nationally and within the seven states with disproportionate rural HIV occurrence, counties were grouped as either mostly urban or mostly or completely rural according to designation by the 2010 U.S. Census.^††^ Rao-Scott chi-square tests were used to compare testing percentages between mostly urban and mostly or completely rural areas in the United States and in the seven states with disproportionate rural HIV occurrence. All estimates were weighted to account for the complex multistage sampling design. HIV diagnosis rates per 100,000 population among persons aged ≥13 years were calculated from HIV diagnoses reported to CDC’s National HIV Surveillance System during 2016–2017 through December 2018; U.S. Census population estimates for 2016 and 2017 were used for the denominators. HIV diagnosis rates and testing percentages were examined together for each of the 50 local jurisdictions as well as urban and rural areas of the seven states to further characterize these areas with respect to their current HIV morbidity and testing coverage; Pearson’s correlation coefficient was used to assess the correlation between these areas’ testing percentages and HIV diagnosis rates. Although BRFSS testing percentages were calculated among those aged ≥18 years, HIV diagnosis rates were calculated among those aged ≥13 years to be consistent with methodology used to identify the jurisdictions accounting for the majority of new HIV diagnoses and because of limited availability of single-year age population estimates at the municipio (county equivalent) level in Puerto Rico. Analyses were performed using SAS (version 9.4; SAS Institute) and SUDAAN (version 11.0; RTI International).

During 2016–2017, 38.9% of adults aged ≥18 years in the United States had ever been tested for HIV (Table 1). Among 15,701 (3.2%) persons with reported recent HIV risk for whom at least annual rescreening is recommended, 64.8% were ever tested, and 29.2% were tested in the past year. Among all adults, the percentage ever tested (46.9%) was higher among residents of the 50 local jurisdictions that accounted for the majority of diagnoses of HIV infection among persons aged ≥13 years than was the percentage ever tested (35.5%) in the seven states with disproportionate rural HIV occurrence. Among persons with reported HIV risk, the percentage tested in the past year (34.3%) in the 50 local jurisdictions was also higher than that in the seven states (26.2%). Among all adults in these seven states, 32.1% of those residing in mostly rural areas and 37.2% of those residing in mostly urban areas had ever been tested. Among persons with reported HIV risk in these states, 18.4% of those residing in rural areas and 29.0% of those residing in urban areas were tested in the past year.

**TABLE 1:** Ever and past-year testing for human immunodeficiency virus (HIV) among adults aged ≥18 years, by urban-rural classification* — Behavioral Risk Factor Surveillance System, United States, 50 local jurisdictions and seven states,^†^ 2016–2017

Status	Total weighted % (95% CI)	Mostly urban counties weighted % (95% CI)	Mostly or completely rural counties weighted % (95% CI)	p-value^§^
Ever tested for HIV				
United States	**38.9 (38.7–39.2)**	40.1 (39.8–40.4)	32.0 (31.5–32.4)	<0.001
50 local jurisdictions	**46.9 (46.3–47.5)**	46.9 (46.3–47.5)	N/A	N/A
Seven states	**35.5 (35.0–36.0)**	37.2 (36.6–37.8)	32.1 (31.3–32.9)	<0.001
**Tested for HIV in the past year**				
United States	**10.1 (9.9–10.2)**	10.6 (10.4–10.8)	6.7 (6.4–7.0)	<0.001
50 local jurisdictions	**14.5 (14.0–14.9)**	14.5 (14.0–14.9)	N/A	N/A
Seven states	**9.3 (8.9–9.6)**	10.1 (9.7–10.5)	7.6 (7.2–8.1)	<0.001
**Tested for HIV in the past year among those with reported HIV risk**				
United States	**29.2 (27.9–30.6)**	30.2 (28.8–31.8)	20.9 (17.7–24.4)	<0.001
50 local jurisdictions	**34.3 (31.3–37.3)**	34.3 (31.3–37.3)	N/A	N/A
Seven states	**26.2 (23.4–29.3)**	29.0 (25.5–32.8)	18.4 (14.5–23.2)	<0.001

**Abbreviations:** CI = confidence interval; N/A = not applicable.

* Urban and rural classifications were derived from 2010 U.S. Census. Counties with <50% of the population residing in areas defined as rural were classified as urban counties. Counties with ≥50% of the population residing in areas defined as rural were classified as rural counties.

^†^ The 50 local jurisdictions (48 counties, the District of Columbia, and San Juan, Puerto Rico) accounted for the majority of new HIV diagnoses, and the seven states (Alabama, Arkansas, Kentucky, Mississippi, Missouri, Oklahoma, and South Carolina) experienced disproportionate occurrence of HIV in rural areas, as identified from HIV diagnoses made during 2016–2017 and reported to the National HIV Surveillance System through June 2018. Diagnosis data from 2017 were considered preliminary.

^§^ Rao-Scott chi-square p-values compare testing estimates between mostly urban counties and mostly or completely rural counties

Testing percentages varied widely by jurisdiction (Table 2). Among the 50 local jurisdictions, the percentage of persons aged ≥18 years ever tested ranged from 36.5% in Maricopa County, Arizona, to 70.7% in the District of Columbia; the percentage tested in the past year (independent of reported recent HIV risk) ranged from 8.1% in Alameda County, California, to 31.3% in Bronx County, New York. Testing percentages were generally low in both urban and rural areas of the seven states with disproportionate rural HIV occurrence. Among the 50 local jurisdictions and seven states, the percentage of persons aged ≥18 years ever tested for HIV generally increased with increasing HIV diagnosis rate among persons aged ≥13 years (r = 0.71; p<0.01) (Figure). Most of the 50 local jurisdictions had higher testing percentages and diagnosis rates than did the seven states.

**TABLE 2:** Ever and past-year testing for human immunodeficiency virus (HIV) among adults aged ≥18 years — Behavioral Risk Factor Surveillance System, 50 local jurisdictions and seven states,* 2016–2017

Jurisdiction	No. of respondents^†^	Ever tested for HIV weighted % (95% CI)	Tested in past year for HIV weighted % (95% CI)
**50 local jurisdictions that accounted for the majority of new HIV diagnoses**			
**Arizona**			
Maricopa County	11,130	36.5 (35.1–37.9)	8.4 (7.6–9.3)
**California**			
Alameda County	740	37.7 (33.3–42.3)	8.1 (5.8–11.2)
Los Angeles County	3,479	43.6 (41.3–45.9)	13.4 (11.9–15.0)
Orange County	1,206	39.8 (36.1–43.6)	10.9 (8.7–13.6)
Riverside County	920	39.6 (35.7–43.7)	10.3 (8.0–13.1)
Sacramento County	952	42.0 (38.1–46.0)	9.1 (7.1–11.7)
San Bernardino County	859	43.0 (38.8–47.2)	12.7 (10.1–15.8)
San Diego County	1,543	45.5 (42.3–48.7)	14.3 (12.1–16.8)
San Francisco County	442	51.8 (45.3–58.3)	14.9 (11.3–19.3)
**District of Columbia**	7,125	70.7 (69.2–72.1)	26.4 (25.0–27.8)
**Florida**			
Broward County	923	54.0 (49.4–58.5)	19.0 (15.6–23.0)
Duval County	1,502	57.0 (52.9–61.0)	20.3 (16.7–24.4)
Hillsborough County	1,148	52.7 (48.4–56.9)	15.3 (12.3–18.8)
Miami-Dade County	1,377	56.7 (52.4–60.9)	18.5 (15.2–22.3)
Orange County	1,301	48.6 (44.6–52.7)	14.9 (12.2–18.1)
Palm Beach County	911	45.5 (40.9–50.1)	11.1 (8.4–14.4)
Pinellas County	890	41.0 (36.4–45.8)	12.4 (9.0–16.7)
**Georgia**			
Cobb County	576	43.7 (38.9–48.7)	10.1 (7.4–13.6)
DeKalb County	603	57.1 (52.2–61.9)	19.5 (15.6–24.0)
Fulton County	967	56.9 (53.2–60.5)	19.7 (16.8–23.1)
Gwinnett County	563	43.2 (38.4–48.2)	11.8 (8.9–15.5)
**Illinois**			
Cook County	3,807	41.3 (39.3–43.2)	13.5 (12.2–14.9)
**Indiana**			
Marion County	3,248	45.4 (42.9–47.9)	13.0 (11.2–14.9)
**Louisiana**			
East Baton Rouge Parish	664	49.7 (44.3–55.2)	17.0 (13.2–21.6)
Orleans Parish	423	58.2 (51.7–64.4)	24.0 (18.2–31.1)
**Maryland**			
Baltimore City	1,735	62.4 (59.2–65.6)	25.3 (22.3–28.6)
Montgomery County	3,366	44.1 (41.7–46.5)	10.6 (9.2–12.3)
Prince George’s County	2,598	56.3 (53.4–59.1)	22.4 (20.1–24.9)
**Massachusetts**			
Suffolk County	1,495	48.6 (44.7–52.5)	15.2 (12.5–18.2)
**Michigan**			
Wayne County	2,906	45.3 (43.1–47.5)	14.1 (12.5–15.8)
**Nevada**			
Clark County	2,770	40.7 (38.5–42.9)	10.9 (9.5–12.4)
**New Jersey**			
Essex County	1,581	55.0 (51.0–59.0)	17.3 (14.4–20.6)
Hudson County	905	50.2 (45.4–54.9)	15.8 (12.5–19.6)
**New York**			
Bronx County	1,094	70.0 (66.4–73.4)	31.3 (28.1–34.8)
Kings County	2,030	57.0 (54.3–59.7)	21.6 (19.4–23.9)
New York County	1,782	60.0 (57.0–62.9)	22.0 (19.6–24.6)
Queens County	1,568	52.3 (49.2–55.5)	18.0 (15.7–20.6)
**North Carolina**			
Mecklenburg County	753	47.1 (42.9–51.3)	13.5 (10.8–16.8)
**Ohio**			
Cuyahoga County	1,172	44.2 (40.7–47.9)	11.9 (9.6–14.6)
Franklin County	1,749	42.3 (39.4–45.1)	10.1 (8.5–12.1)
Hamilton County	912	41.6 (37.7–45.7)	11.3 (8.9–14.3)
**Pennsylvania**			
Philadelphia County	1,399	57.5 (54.2–60.7)	21.4 (18.8–24.3)
**Puerto Rico**			
San Juan Municipio	1,042	57.2 (52.7–61.6)	17.0 (14.0–20.5)
**Tennessee**			
Shelby County	717	53.4 (49.0–57.8)	22.8 (18.9–27.3)
**Texas**			
Bexar County	784	45.1 (39.9–50.5)	13.7 (10.2–18.1)
Dallas County	623	44.2 (38.7–49.8)	14.4 (10.7–19.2)
Harris County	1,214	45.9 (41.9–50.0)	13.2 (10.8–16.2)
Tarrant County	740	46.0 (40.8–51.4)	11.6 (8.3–16.0)
Travis County	1,855	50.2 (46.2–54.2)	12.3 (9.9–15.3)
**Washington**			
King County	6,101	39.4 (37.9–40.9)	8.4 (7.5–9.3)
**Seven states with disproportionate HIV occurrence in rural counties**			
**Alabama, total**	**12,098**	**39.4 (38.3–40.6)**	**11.0 (10.2–11.8)**
Urban counties	7,442	40.8 (39.4–42.3)	12.1 (11.1–13.2)
Rural counties	4,656	36.8 (34.8–38.8)	8.8 (7.6–10.2)
**Arkansas, total**	**9,268**	**33.7 (31.9–35.6)**	**9.1 (7.9–10.4)**
Urban counties	5,206	35.8 (33.4–38.3)	10.6 (8.9–12.5)
Rural counties	4,062	30.9 (28.3–33.6)	7.1 (5.7–8.8)
**Kentucky, total**	**16,937**	**33.8 (32.6–34.9)**	**7.2 (6.6–7.9)**
Urban counties	8,887	36.3 (34.7–38.0)	8.0 (7.1–9.0)
Rural counties	8,050	29.9 (28.4–31.4)	6.0 (5.3–6.9)
**Mississippi, total**	**8,984**	**40.2 (38.7–41.7)**	**12.7 (11.6–13.9)**
Urban counties	4,207	44.3 (42.2–46.5)	14.3 (12.7–16.1)
Rural counties	4,777	35.4 (33.4–37.4)	10.9 (9.5–12.4)
**Missouri, total**	**13,446**	**34.3 (33.1–35.5)**	**8.3 (7.5–9.1)**
Urban counties	9,031	36.4 (34.8–37.9)	9.3 (8.4–10.4)
Rural counties	4,415	29.1 (27.1–31.3)	5.6 (4.5–6.8)
**Oklahoma, total**	**11,952**	**29.7 (28.6–30.9)**	**6.8 (6.2–7.6)**
Urban counties	7,365	30.7 (29.2–32.2)	7.4 (6.5–8.4)
Rural counties	4,587	27.8 (26.0–29.7)	5.7 (4.8–6.9)
**South Carolina, total**	**19,983**	**37.4 (36.4–38.3)**	**10.6 (9.9–11.3)**
Urban counties	14,201	37.7 (36.5–38.8)	10.5 (9.8–11.4)
Rural counties	5,782	36.1 (34.3–38.0)	10.9 (9.6–12.4)

**Abbreviation:** CI = confidence interval.

* Urban and rural classifications were derived from 2010 U.S. Census. Counties with <50% of the population residing in areas defined as rural were classified as urban counties. Counties with ≥50% of the population residing in areas defined as rural were classified as rural counties. The 50 local jurisdictions (48 counties, the District of Columbia, and San Juan, Puerto Rico) accounted for the majority of new HIV diagnoses, and the seven states (Alabama, Arkansas, Kentucky, Mississippi, Missouri, Oklahoma, and South Carolina) experienced disproportionate occurrence of HIV in rural areas, as identified from HIV diagnoses made during 2016–2017 and reported to the National HIV Surveillance System through June 2018. Diagnosis data from 2017 were considered preliminary.

**^†^** Number of respondents with “yes” or “no” response to question about ever testing for HIV.

**FIGURE:**
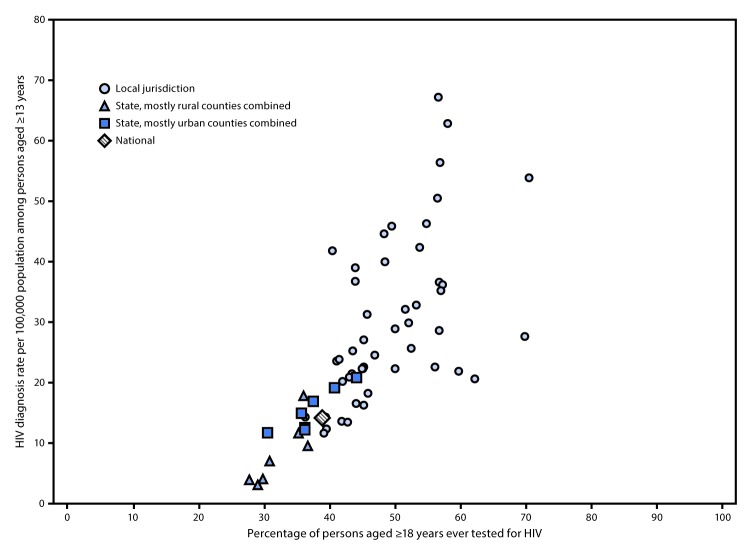
Percentage of adults aged ≥18 years ever tested for human immunodeficiency virus (HIV) infection and HIV diagnosis rate* among persons aged ≥13 years — Behavioral Risk Factor Surveillance System and National HIV Surveillance System (NHSS), 50 local jurisdictions accounting for the majority of new HIV diagnoses and seven states with disproportionate occurrence of HIV in rural areas,^†^ 2016–2017^§^ * HIV diagnosis rates per 100,000 population among persons aged ≥13 years during 2016–2017 were calculated from HIV diagnoses reported to NHSS through December 2018 and Census population estimates for 2016 and 2017. ^†^ The 50 local jurisdictions (48 counties, the District of Columbia, and San Juan, Puerto Rico) accounted for the majority of new HIV diagnoses, and the seven states (Alabama, Arkansas, Kentucky, Mississippi, Missouri, Oklahoma, and South Carolina) experienced disproportionate occurrence of HIV in rural areas, as identified from HIV diagnoses made during 2016–2017 and reported to NHSS through June 2018. Diagnosis data from 2017 were considered preliminary/ ^§^ Pearson’s correlation coefficient = 0.71; p<0.01.

## Discussion

In this analysis, <40% of the U.S. adult population had ever been tested for HIV. Jurisdictions with the highest rates of diagnosis of HIV infection among persons aged ≥13 years generally had higher testing percentages. The converse was also true. Ever testing for HIV was lower in rural areas of the seven states with disproportionate rural HIV occurrence, compared with that in urban areas of these states, the 50 local jurisdictions with the majority of diagnoses of HIV infection, and the United States nationally. Although past-year HIV testing was higher among persons with reported recent HIV risk than among those without such risk, the percentage tested in the past year was far below the 100% coverage recommended for this group (*1*,*2*). These findings demonstrate missed opportunities to fully implement HIV screening recommendations in the 57 jurisdictions that will serve as the initial geographic focus of the Ending the HIV Epidemic initiative. The observed variability in both ever and past-year testing by jurisdiction highlights the need for screening strategies that are tailored to local needs. BRFSS is likely the only annual survey with a sufficient sample size to provide jurisdiction-level estimates of HIV testing to monitor long-term progress toward increasing screening coverage in the United States.

HIV screening strategies will likely need to be locally tailored and novel to reach segments of the population that have not been reached by previous efforts. Examples of novel or promising approaches to increase access to HIV testing include routinizing HIV screening in health care settings, integrating HIV screening with sexual health screenings, scaling up partner notification and other strategies (using social network strategy^§§^ or mobile applications) that offer screening of the social and sexual networks of persons seeking HIV screening, promoting pharmacist-led screening^¶¶^ as well as screening in other alternative clinical settings such as urgent care, and mass distribution of HIV self-tests*** (*6*–*10*). Further efforts will be needed to identify which approaches are most effective in increasing access to HIV testing in various settings and jurisdictions with different baseline needs. Early diagnosis and effective treatment that suppresses HIV replication not only minimize immune system damage and reduce individual morbidity and mortality but also reduce the risk for transmission to others.^†††^ Delayed diagnosis limits these benefits. HIV screening is a critical entry point to a range of HIV prevention and treatment options. For persons at ongoing risk for HIV infection exposure, annual screening also offers the opportunity to discuss options to reduce risk, including HIV preexposure prophylaxis.^§§§^

The findings in this report are subject to at least six limitations. First, because the proportion of respondents reporting recent HIV risk was small, testing percentages for this group could not be reported separately in the 57 jurisdictions. Second, self-reported data might be subject to social desirability and recall biases, which might have led to over- or underestimation of testing. Third, BRFSS response rates were low; however, the response rates are comparable with those of other national landline and cellular telephone surveys, and survey weights were designed to ensure generalizable findings. Fourth, the measure of HIV-related risk did not include every behavior that might increase risk for HIV infection, such as unprotected sex with a partner who is known to have HIV or whose HIV status is unknown. Fifth, the assessment of HIV diagnosis rates and HIV testing percentages relied on disparate age ranges (≥13 years and ≥18 years, respectively). Finally, this analysis included data from surveys conducted during 2016–2017 and HIV diagnoses that occurred during the same period. These are the most current data available for these measures but represent a delayed cross-section of the current state of HIV testing and diagnoses for 2019. To monitor progress toward national goals, closer to real-time reporting of select HIV testing activities might be needed.

HIV screening remains suboptimal for persons residing in the 57 jurisdictions that will constitute the initial geographic focus of the Ending the HIV Epidemic initiative. These data provide a baseline from which to measure changes in screening in these jurisdictions and other parts of the United States over time. To achieve national goals and end the HIV epidemic in the United States, innovative and novel screening approaches might be needed to reach segments of the population that have never been tested for HIV.

**Summary**

**What is already known about this topic?**

Rates of screening for human immunodeficiency virus (HIV) in the United States are low.

**What is added by this report?**

This analysis of national survey data found that <40% of U.S. adults had ever been tested for HIV, and testing rates varied among jurisdictions comprising the initial focus of the Ending the HIV Epidemic initiative. Within these jurisdictions, rural areas had lower testing percentages and lower HIV diagnosis rates than did urban areas.

**What are the implications for public health practice?**

Novel HIV screening strategies tailored to meet local needs might be needed to reach segments of the population that have never been tested for HIV and achieve national goals to end the HIV epidemic in the United States.

## Footnotes

* https://www.hiv.gov/federal-response/ending-the-hiv-epidemic/overview.

† The 50 local jurisdictions and seven states were identified from diagnoses made during 2016–2017 reported to CDC’s National HIV Surveillance System through June 2018. Diagnosis data from 2017 were considered preliminary (https://files.hiv.gov/s3fs-public/ending-the-hiv-epidemic-flyer.pdf). A list of the 50 local jurisdictions and seven states is available in Table 2 of this report and at https://files.hiv.gov/s3fs-public/Ending-the-HIV-Epidemic-Counties-and-Territories.pdf.

§ https://www.cdc.gov/brfss/index.html.

¶ https://www.cdc.gov/brfss/annual_data/2016/pdf/2016-sdqr.pdf.

** https://www.cdc.gov/brfss/annual_data/2017/pdf/2017-sdqr-508.pdf.

†† https://www.census.gov/programs-surveys/geography/guidance/geo-areas/urban-rural.html.

§§ https://effectiveinterventions.cdc.gov/en/care-medication-adherence/group-4/social-network-strategy-for-hiv-testing-recruitment.

¶¶ https://effectiveinterventions.cdc.gov/en/hiv-testing/group-1/hiv-testing-in-retail-pharmacies.

*** https://www.cdc.gov/hiv/testing/hometests.html.

††† https://www.cdc.gov/hiv/risk/art/index.html.

§§§ https://www.cdc.gov/hiv/risk/prep/index.html.

## References

[1] Branson BM, Handsfield HH, Lampe MA, . Revised recommendations for HIV testing of adults, adolescents, and pregnant women in health-care settings. MMWR Recomm Rep 2006;55(No. RR-14). 16988643

[2] DiNenno EA, Prejean J, Irwin K, Recommendations for HIV screening of gay, bisexual, and other men who have sex with men—United States, 2017. MMWR Morb Mortal Wkly Rep 2017;66:830–2. 10.15585/mmwr.mm6631a3 28796758 PMC5687782

[3] Pitasi MA, Delaney KP, Oraka E, Interval since last HIV test for men and women with recent risk for HIV infection—United States, 2006–2016. MMWR Morb Mortal Wkly Rep 2018;67:677–81. 10.15585/mmwr.mm6724a2 29927906 PMC6013085

[4] Dailey AF, Hoots BE, Hall HI, Vital signs: human immunodeficiency virus testing and diagnosis delays—United States. MMWR Morb Mortal Wkly Rep 2017;66:1300–6. 10.15585/mmwr.mm6647e1 29190267 PMC5708685

[5] Fauci AS, Redfield RR, Sigounas G, Weahkee MD, Giroir BP. Ending the HIV epidemic: a plan for the United States. JAMA 2019;321:844–5. 10.1001/jama.2019.1343 30730529

[6] Sullivan PS, Lyons MS, Czarnogorski M, Branson BM. Routine screening for HIV infection in medical care settings: a decade of progress and next opportunities. Public Health Rep 2016;131(Suppl 1):1–4. 10.1177/00333549161310S101 26862224 PMC4720600

[7] Golden MR, Katz DA, Dombrowski JC. Modernizing field services for human immunodeficiency virus and sexually transmitted infections in the United States. Sex Transm Dis 2017;44:599–607. 10.1097/OLQ.0000000000000652 28876325 PMC5637519

[8] Kachur R, Hall W, Coor A, Kinsey J, Collins D, Strona FV. The use of technology for sexually transmitted disease partner services in the United States: a structured review. Sex Transm Dis 2018;45:707–12. 10.1097/OLQ.0000000000000864 29771868 PMC6546166

[9] Weidle PJ, Lecher S, Botts LW, HIV testing in community pharmacies and retail clinics: a model to expand access to screening for HIV infection. J Am Pharm Assoc 2014;54:486–92. 10.1331/JAPhA.2014.14045 25216878 PMC4698873

[10] Katz DA, Golden MR, Hughes JP, Farquhar C, Stekler JD. HIV self-testing increases HIV testing frequency in high-risk men who have sex with men: a randomized controlled trial. J Acquir Immune Defic Syndr 2018;78:505–12. 10.1097/QAI.0000000000001709 29697595 PMC6037557

